# Integrative Taxonomy Reveals New Insights into the Species Validity of the *Neocaridina davidi*-*N. denticulata*-*N. heteropoda* Complex and Mitogenomic Phylogeny of Caridean Shrimps

**DOI:** 10.3390/cimb46110729

**Published:** 2024-10-31

**Authors:** Mei Yang, Xiaodong Cui, Xinzheng Li, Dong Dong, Xianjiang Kang, Zhibin Gan

**Affiliations:** 1 Department of Marine Organism Taxonomy & Phylogeny, Institute of Oceanology, Chinese Academy of Sciences, Qingdao 266071, China; yangmei@qdio.ac.cn (M.Y.); lixzh@qdio.ac.cn (X.L.); dongd@qdio.ac.cn (D.D.); 2 College of Life Sciences, Hebei University, Baoding 071002, China; cxd20181666@163.com; 3 Laboratory for Marine Biology and Biotechnology, Qingdao Marine Science and Technology Center, Qingdao 266237, China; 4 University of Chinese Academy of Sciences, Beijing 100049, China

**Keywords:** *Neocaridina*, integrative taxonomy, morphology, mitogenome, synonym, genetic distances, phylogeny

## Abstract

The genus *Neocaridina*, originating from East Asia and representing a small-size landlocked shrimp group of the family Atyidae, is an important group of ornamental shrimps and plays significant ecological roles in their natural habitats. Owing to the considerable variability of the taxonomic characters it employed, *Neocaridina* is constantly under revision, and the validation of several species is currently questionable. In the present study, several *Neocaridina* shrimps were collected from the Baiyangdian drainage area. Through morphological examination, they exhibited delicately diagnostical differences in the dactyli of the third pereiopod and the endopod of the first and second pleopod and were classified into morph A, morph B and morph C. According to the literature description, morph A and morph C were identified as *N. denticulata denticulata* and *N. denticulata sinensis*, respectively. Among them, morph B presents an intermediate state between morph A and morph C. Subsequently, we determined the mitogenomes of morph A, morph B and morph C. Based on the morphological characteristics, genetic variation and phylogenetic tree, we contend that *N. davidi*, *N. d. denticulata*, *N. d. sinensis* and *N. heteropoda* should belong to the same species, and we propose retaining the name *N. denticulata*. The reconstructed mitogenomic phylogeny indicated that the monophyly of several genera within Atyidae has been challenged, suggesting that the established classification of Atyidae requires substantial taxonomic revision at all taxonomic levels. Furthermore, the tree’s topologies supported Atyidae at a deeper base within Caridea. More comprehensive taxon sampling is still needed to resolve the explicit internal relationships among Caridea.

## 1. Introduction

Atyidae is the most diversified caridean family occurring in freshwaters around the world, comprising close to 500 species and exhibiting a wide range of breeding and feeding strategies in various habitats [1,2]. The genus *Neocaridina* Kubo, 1938 is a small size landlocked shrimp group distributed in East Asia and comprises more than 20 species [3,4]. These *Neocaridina* shrimps play important ecological roles in freshwater ecosystems (e.g., benthic community composition, decomposition of organic matter and relevant transferring and nutrient cycling) and serve as crucial prey species for macroinvertebrates, fish and water birds [5,6]. In addition, due to the rich and varied integument coloration, *Neocaridina* has great success in the aquarium market as an ornamental shrimp group [7]. In contrast to the significant ecological value in their natural habitats and active utilizations, the taxonomy of some *Neocaridina* taxa has been controversial and the validation of a few species is currently questionable [3,8,9,10].

Historically, the swamp shrimp *Neocaridina denticulata* De haan, 1844 had been mainly divided into two subspecies, *N. d. denticulata* and *N. d. sinensis*, according to their different morphological characters and geographical distributions [11]. The former is endemic in the main islands of Japan, while the latter has been mainly present in East and Central China and Taiwan [8,11], as well as in Hawaii as an introduced species [12]. In 2002, Liang described a new species, *N. heteropoda*, from Zhejiang Province, China, and referred some of Cai’s [8] *N. d. sinensis* to this new species [13]. Liang [3,13] considered the characters distinguishing *N. d. denticulata* and *N. d. sinensis* to be slight and not reliable, and treated *N. d. sinensis* from China and Taiwan as a synonym of *N. d. denticulata*, and referred the form introduced to Hawaii as *N. heteropoda*. De Grave and Fransen [14] also suggested *N. d. sinensis* is a synonym of *N. d. denticulata*. Moreover, both Shih et al. and Klotz et al. [4,9,10] suggested that *N. d. sinensis* was synonymous with *N. davidi*. Considering the taxonomic complexity within the species complex of *N. davidi*-*N. denticulata*-*N. heteropoda*, several morphological and molecular analyses have been conducted, but no conclusive results have been obtained so far [4,9,15,16].

Furthermore, some controversy still exists around the evolutionary position of Atyidae within Caridea. Based on mitochondrial and nuclear gene markers, Bracken et al. [17] suggested that Atyidae represents the basal lineage of Caridea. However, Tan et al. found Atyidae as the sister clade of Alvinocarididae within Caridea [18]. More recently, Sun et al. [19] indicated that Atyidae and (((Acanthephyridae + Oplophoridae) + Alvinocarididae) + Nematocarcinidae) are clustered together. Thus, it can be seen that the systematic status among different families within Caridea has also shown inconsistencies in previous systematic studies [19,20,21,22].

During the past few decades, the rapid progress in modern molecular biology has significantly contributed to the advancement of research in molecular systematics. By studying molecular sequences to investigate the phylogenetic relationships among species and families, it becomes possible to effectively overcome the limitations of traditional taxonomy and address numerous contentious issues in the fields of classification and systematic evolution. The mitochondrial genome (mitogenome) is characterized by small genome size, rich genetic information, rare recombination, and relatively fast evolutionary rates [23,24] and it has been widely used for species identification, population genetics, phylogenetic relationships and phylogeography, showing promising potential in settling systematic disputes [25,26,27,28]. Moreover, gene order rearrangement of the mitogenome could offer additional valuable evolutionary information [29,30,31]. In the mitogenomes of Decapoda, gene rearrangement is commonly observed [30,32] and novel mitochondrial gene orders have been reported in caridean shrimps [18,21,22].

Hitherto, more than thirty complete mitogenomes of Atyidae have been published and distributed within nine genera (*Atyopsis*, *Caridina*, *Halocaridina*, *Halocaridinides*, *Neocaridina*, *Paratya*, *Stygiocaris*, *Typhlatya*, *Typhlopatsa*), and the phylogenetic relationships based on mitogenomes within Atyidae are relatively scarce. Furthermore, although *Neocaridina* shrimps have significant ecological and economic value, research on their taxonomic status based on morphological characteristics and molecular data is extremely limited. Recently, three groups of *Neocaridina denticulata* shrimps (morph A, morph B and morph C) were collected from the Baiyangdian drainage area, Hebei Province, exhibiting certain morphological differences among them. Based on these specimens and their complete mitogenomes, we present: (i) an assessment of the interspecific and intraspecific mitogenomic divergence of the *Neocaridina* shrimps; (ii) a discussion on the distinctiveness of *N. denticulata*, *N. heteropoda*, *N. davidi* and related subspecies based on morphological and phylogenetic analysis; and (iii) an evaluation of the systematic status of Atyidae and other families of Caridea based on mitogenomic analyses.

## 2. Materials and Methods

### 2.1. Specimen Collection and Morphological Examination

**Figure 1:**
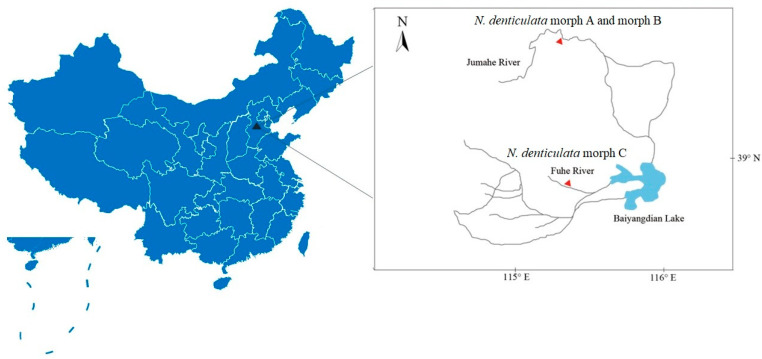
The sampling locations of *Neocaridina denticulata* (morph A, morph B and morph C) in this study.

Mature males of three groups of *N. denticulata* shrimps (morph A, morph B and morph C) were collected from the freshwater of Baiyangdian drainage area, Hebei Province, China (Figure 1): morph A and morph B from the Jumahe River (39.66° N, 115.47° E) and morph C from the Fuhe River (38.85° N, 115.46° E). All specimens were immediately preserved in 95% ethanol and deposited at the Marine Biological Museum, Chinese Academy of Sciences (MBMCAS), Qingdao, China. Specimens were dissected with a stereomicroscope (SMZ1500, Nikon, Tokyo, Japan) and photographed with a microscope (AZ100, Nikon, Tokyo, Japan).

### 2.2. Mitogenome Assembly and Annotation

Total genomic DNA of the specimens was extracted using E.Z.N.A Tissue DNA kit (OMEGA, Shanghai, China) following the manufacturer’s protocol. The paired-end libraries with an insert size of 450 bp were constructed from total genomic DNA using a TruSeq™ Nano DNA Sample Prep Kit (Illumina, San Diego, CA, USA) and then sequenced on the Illumina HiSeq 4000 platform (2 × 150 bp paired-end reads). Quality control on the paired-end raw data was performed using Trimmomatic 0.39 [33], and it involved eliminating adapters, duplicated sequences, reads with a quality score below 20 and reads containing ≥10% of uncalled bases. The resulting clean data was assembled de novo by GetOrganelle [34]. The contigs identified as mitogenome sequences were aligned with the available complete mitogenomes of Atyidae and manually examined for repeats, thus circular mitochondrial DNA was established.

The MITOS webserver [24] was used to annotate the assembled mitogenomes with default settings and invertebrate mitochondrial code. Boundaries of the protein-coding genes (PCGs) were further identified by the Open Reading Frame Finder (https://www.ncbi.nlm.nih.gov/orffinder/ (accessed on 17 October 2024)), and the exact initiation/termination codon positions were manually determined. Ribosomal RNA (rRNA) genes were confirmed by rRNAmmer webserver [35]. Transfer RNA (tRNA) genes and their secondary structures were further predicted with ARWEN Online services [36]. The newly complete mitogenomes have been deposited in GenBank under accession numbers PQ246621-PQ246623.

The nucleotide composition, codon usage and relative synonymous codon usage (RSCU) were calculated using DnaSP 6.0 [37]. The AT and GC skews were measured with the following formulas: AT skew = (A − T)/(A + T) and GC skew = (G − C)/(G + C) [38]. The Kimura’s 2-parameter genetic distances among atyid shrimps were calculated using MEGA X [39].

### 2.3. Phylogenetic Analysis

The mitogenomic phylogenetic trees were reconstructed based on the three newly determined *N. denticulata* mitogenomes and those of fifty-nine caridean species belonging to ten families, with two species of Dendrobranchiata and two species of Stomatopoda as the outgroups (Table S4). The substitution saturation test of PCGs and rRNA genes was performed by DAMBE 7 [40]. The substitution saturation indexes were significantly lower than the threshold value (Iss < Iss.c), suggesting that the related sequences were little saturated and, therefore, appropriate for phylogenetic analysis [41]. All codons of the *atp8* gene, the 3rd codons of other PCGs and the two rRNA genes were discarded due to high saturation (Figure S1). Consequently, the phylogenetic analyses were carried out using two datasets. The first dataset contained nucleotide sequences of 12 PCGs (except *atp8* since a high saturation was detected for this gene) at the first and second codon positions (without the 3rd codon since a high saturation was detected on this position), and it is referred to as the NT dataset. The second dataset comprised amino acid sequences of 13 PCGs, hence referred to as the AA dataset.

The nucleotide sequences and amino acid sequences of mitochondrial PCGs were aligned separately using MAFFT with default parameters [42]. The ambiguously aligned regions were removed by Gblocks (default settings) [43]. Then, the trimmed alignments were concatenated into a single dataset using PhyloSuite 1.2.3 [44]. The partition schemes and best-fit substitution models for NT and AA data were inferred by PartitionFinder [45] (Table S5). Phylogenetic relationships were inferred using both maximum likelihood (ML) and Bayesian inference (BI) methods for each dataset. The ML tree was constructed in IQ-TREE [46] and node reliability was assessed by 5000 ultrafast bootstrap replicates [47]. Bayesian analysis was performed by MrBayes [48]. The Markov chain Monte Carlo (MCMC) runs for 10,000,000 generations starting from a random tree and sampling every 1000 generations. We monitored the average standard deviation of split frequencies and likelihood values to assess whether the two runs had converged to a stationary distribution. The first 25% of the trees were discarded as burn-in. The remaining trees were used to construct the 50% majority rule consensus tree and the Bayesian posterior probabilities (PP).

### 2.4. Gene Order Analysis

We mapped all patterns of mitochondrial gene orders onto the phylogeny for comparison. All the mitochondrial genes (PCGs, rRNAs and tRNAs) were considered. The putative ancestral state of the pancrustacean gene order pattern and the other mitochondrial gene order patterns were pairwise compared to predicting the gene rearrangement events (e.g., reversals [R], transpositions [T], reverse transpositions [rT], tandem duplication random loss [TDRL]) using Common interval Rearrangement Explorer (CREx), heuristically exploring mitochondrial genome rearrangements based on common intervals [49].

## 3. Results

### 3.1. Morphological Classification

**Figure 2:**
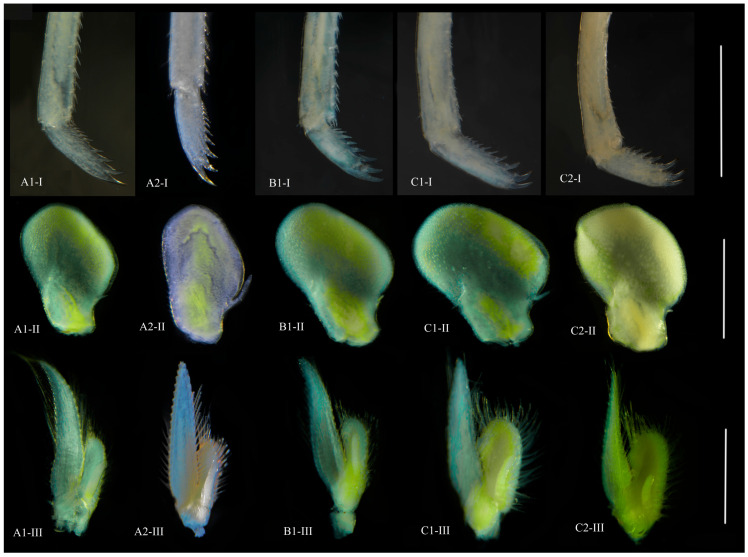
Diagnostic features of the males of N. denticulata (morph A, morph B and morph C); morph A: A1, A2; morph B: B1; morph C: C1, C2; I: dactyli of the third pereiopod; II: endopod of the first pleopod; III: endopod of the second pleopod. Scales: 1000 μm.

The identification among the genus *Neocaridina*, particularly referring to *N. denticulata denticulata*, *N. d. sinensis*, *N. heteropoda*, *N. davidi*, etc., is always confusing [8,9,13], due to the delicately diagnostical differences in the rostrum length, the dactyli of the third pereiopod and the endopod of the first and second pleopod (Figure 2). According to Cai [8], male specimens exhibiting normal third pereiopod dactyli (Figure 2: morph A: A1, A2) were identified as *N. denticulata denticulata*, while those displaying sexual dimorphism in third pereiopod dactyli (the dactyli is conspicuously broad, and the several terminal spines at the dactyli are thick, long and curved and slightly hook-shaped; the females are the opposite) (Figure 2: morph C: C1, C2) were classified as *N. denticulata sinensis*. However, Liang [13] considered the latter to be *N. heteropoda*. In the present study, we collected both the morphs in the Baiyangdian waters (Figure 2: A1, A2, C1, C2), particularly, we also found some specimens with the third pereiopod dactyli exhibiting an intermediate state between morph A and morph C (Figure 2: morph B: B1), indicating the continuous variation of the dactyli of the third pereiopod. Additionally, Liang [3] reclassified the subspecies *N. denticulata* koreana as *N. heteropoda* koreana, citing the length-width ratio of the endopod of the first pleopod as the basis for this reclassification. Here we found that this ratio should also be considered as intraspecific variation (Figure 2: C1-II, C2-II), as well as the length ratio of appendix masculine (Figure 2: B1-III, C2-III).

### 3.2. Organization and Characterization of Mitogenomes

**Figure 3:**
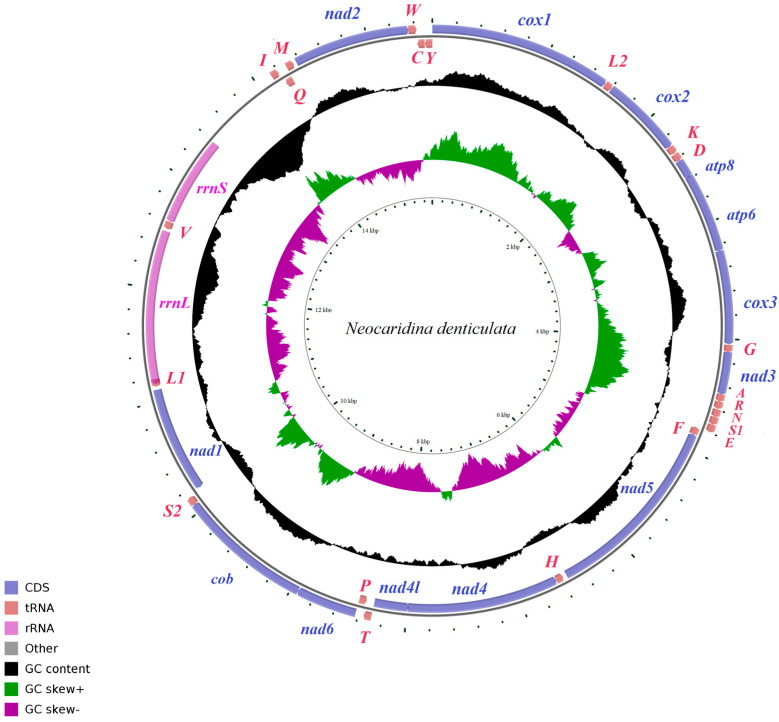
The organization of the mitogenome of *Neocaridina denticulata*. Genes for proteins and rRNAs are shown with standard abbreviations. Genes for tRNAs are represented by a single letter for the corresponding amino acid, with two leucine tRNAs and two serine tRNAs differentiated by numerals.

The mitogenomes of the three *N. denticulata* shrimps (morph A, morph B and morph C) were all circularized from the clean data, with 15,553 (morph A), 15,558 (morph B) and 15,554 bp (morph C) in length, respectively. In the newly sequenced three mitogenomes, 13 protein-coding genes (PCGs), 2 ribosomal RNA genes (rRNAs) and 22 transfer RNA (tRNAs) were detected as typical in most metazoans [50]. Among these genes, 23 genes (9 PCGs and 14 tRNAs) were encoded by the heavy (H) strand, while the remaining 14 genes (4 PCGs, 8 tRNAs and 2 rRNAs) were encoded by the light (L) strand (Figure 3). All PCGs of the three newly sequenced mitogenomes used the typical ATN as initiation codons, and most PCGs have the complete stop codons (TAA and TAG), while the *cox2* and *nad4* ended by a single T nucleotide. The strand position, length and start/stop/anti codon are summarized in Table 1.

At present, the published mitogenome size from 9 Atyidae genera was less than 17,000 bp, with the longest being *Caridina longshan* (16,853 bp) and the shortest being *Typhlatya garciai* (15,318 bp). The variations in the length of Atyidae mitogenomes primarily result from the heterogeneity of the non-coding regions. Furthermore, there exists a noteworthy and robust positive association between the mitogenome size and the length of non-coding regions (ρ = 0.965, *p* < 0.001) (Table S1).

**Figure 4:**
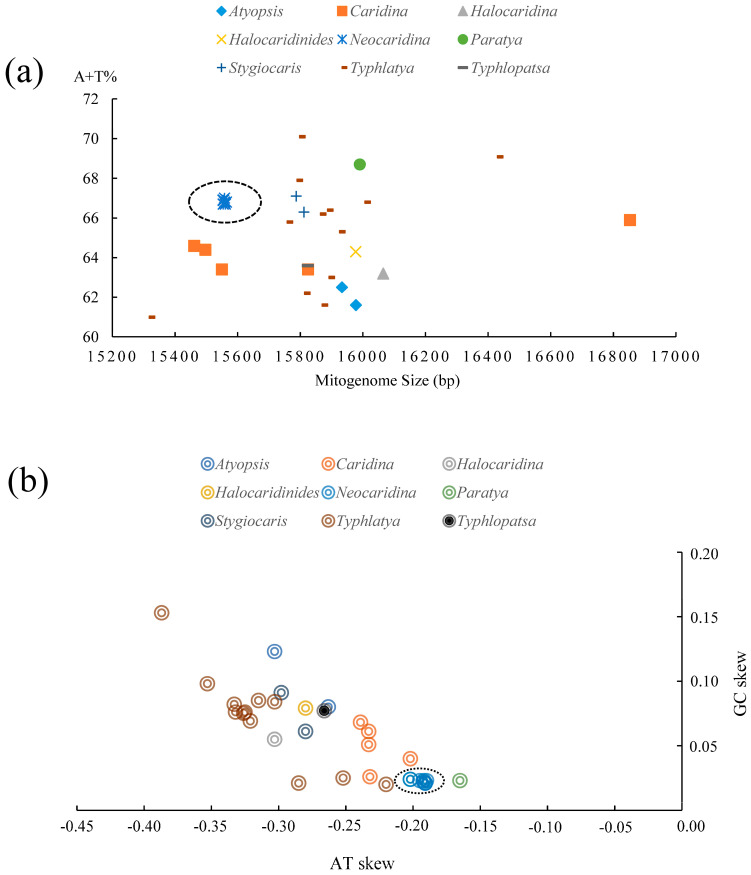
(**a**) Genome size versus A + T content of the Atyidae mitogenomes. Different genera are represented by different shapes and colors. (**b**) AT-skew and GC-skew in the Atyidae mitogenomes. Different genera are represented by different shapes and colors. The black dotted line ovals represent the genus *Neocaridina*.

The A + T content of all available Atyidae mitogenomes ranged from 61% in *Typhlatya garciai* to 70.1% in *T*. *pearsei* (Figure 4a). The values of the AT-skews were all negative for the published Atyidae mitogenomes while the GC-skews were positive, indicating more Ts than As and more Gs than Cs (Figure 4b).

**Figure 5:**
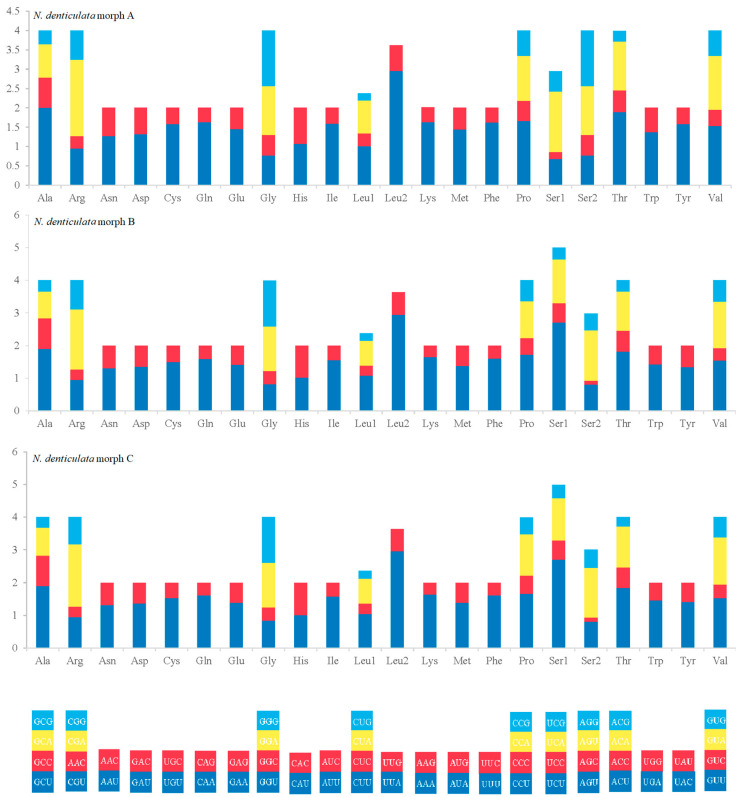
The relative synonymous codon usage (RSCU) of the three new *N. denticulata* mitogenomes (morph A, morph B and morph C). The numbers to the left refer to RSCU values. Codon families are provided on the X-axis.

Among the PCGs of three newly sequenced *N. denticulata* mitogenomes, the Leucine, Serine and Isoleucine were the most frequently used amino acids, accounting for about one-third of the total (Table S2). The relative synonymous codon usage (RSCU) analysis showed that the NNA and NNU were usually higher than 1 (Figure 5), and reflected a bias towards the usage of A and T at the third codon position, which was similar to the biases that exist in most metazoans [25,51].

### 3.3. Genetic Distances

**Figure 6:**
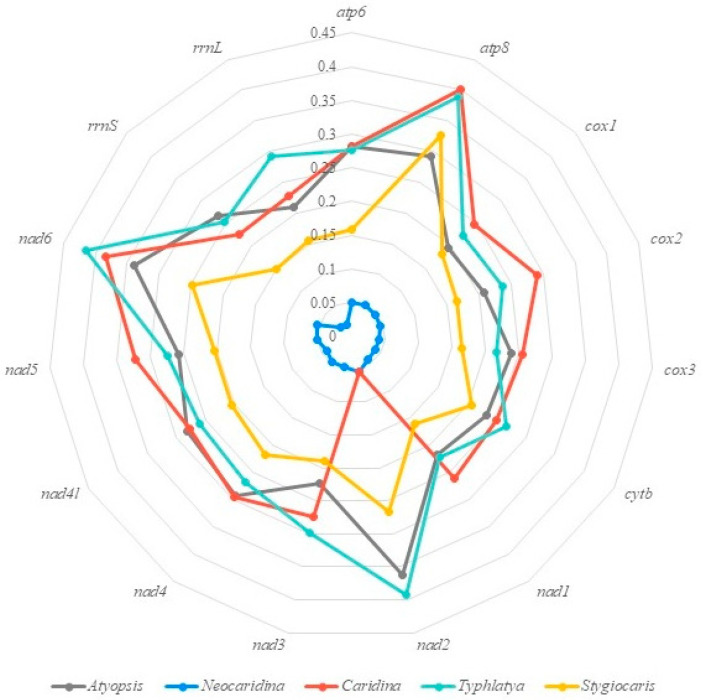
Kimura’s 2-parameter pair-wise genetic distances of 13PCGs, 12S rRNA and 16S rRNA sequences based on the published mitogenome data of Atyidae.

Based on the published mitogenome data, we calculated the genetic divergence of 13PCGs, 12S rRNA (*rrnS*) and 16S rRNA (*rrnL*), respectively, within different genera and species in the Atyidae family. The results demonstrated that the average intrageneric genetic divergence of *Neocaridina* was 4.36%, which was significantly lower than the values of four other genera (*Atyopsis* 26%, *Caridina* 27.11%, *Stygiocaris* 20.01% and *Typhlatya* 28.59%) (Figure 6). Within the genus *Neocaridina*, the *nad2* exhibits the largest (5.5%) genetic distance, while the 16S rRNA has the smallest (1.8%) (Table S3).

**Figure 7:**
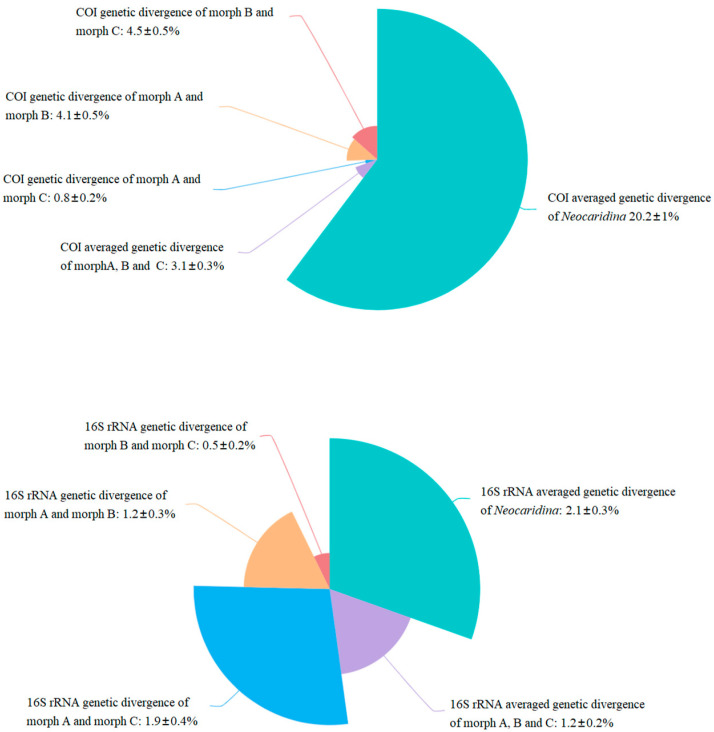
The average genetic distances of COI and 16S rRNA gene sequences within the *Neocaridina* genus vs. the genetic distance of COI and 16S rRNA among the three newly determined *N. denticulata*.

Furthermore, through screening the COI and 16S rRNA gene sequences of *Neocaridina* in the GenBank database, we conducted an analysis of the genetic distance of COI and 16S rRNA within the genus. The average intrageneric genetic divergence of COI and 16S rRNA was 20.2 ± 1% and 2.1 ± 0.3%, respectively. Meanwhile, the average genetic divergence of COI and 16S rRNA for the three new *N. denticulata* mitogenomes (morph A, morph B and morph C) were 3.1 ± 0.3% and 1.2 ± 0.2%, respectively (Figure 7), which were significantly lower than the interspecific genetic distances within the *Neocaridina* genus.

### 3.4. Phylogenetic Analysis

**Figure 8:**
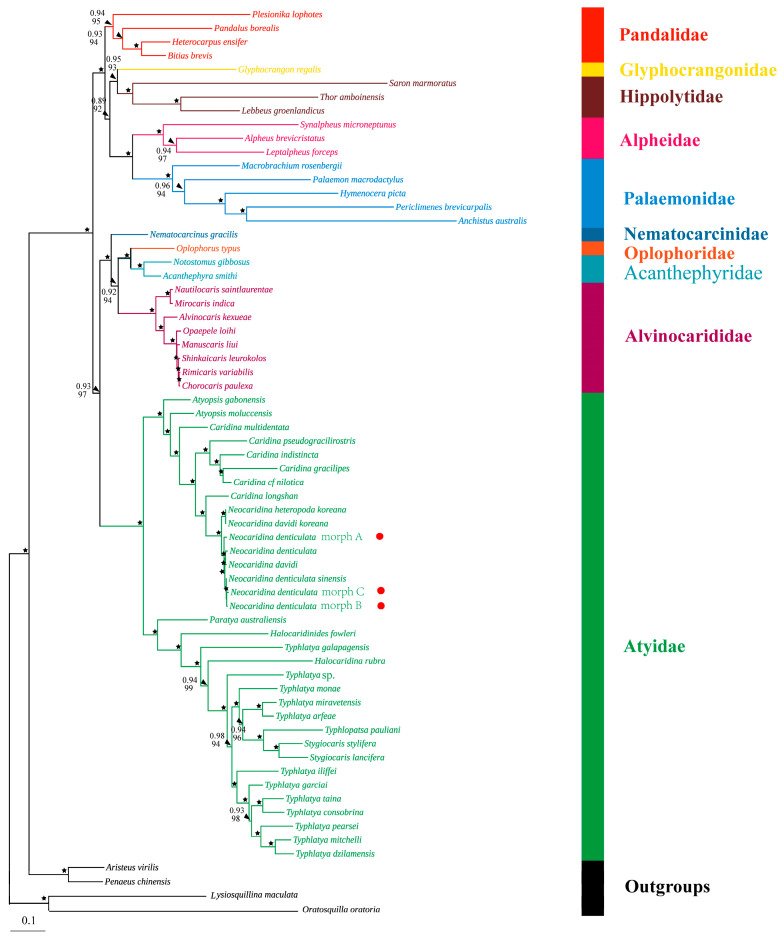
The phylogenetic tree of Caridea inferred from ML and BI methods based on the nucleotide sequences of the first and second codon positions of 12 PCGs (except for the *atp8* gene). The Bayesian posterior bootstrap probability (the number above) and the bootstrap probability (the number below) were shown at each node. The black asterisk (★) indicates both posterior probabilities and bootstrap values ≥ 0.95/95 for the nodes.

**Figure 9:**
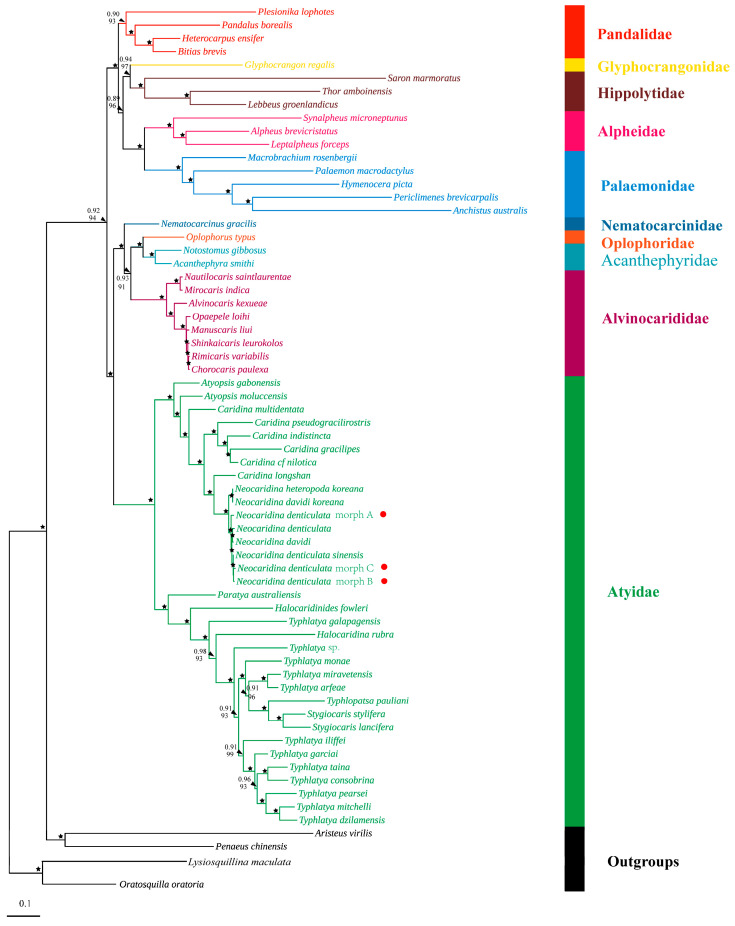
Phylogenetic tree inferred from ML and BI methods based on the amino acid sequences of the 13 PCGs. The Bayesian posterior bootstrap probability (the number above) and the bootstrap probability (the number below) were shown at each node. The black asterisk (★) indicates both posterior probabilities and bootstrap values ≥ 0.95/95 for the nodes.

To obtain reliable phylogenetic results, substitution saturation tests were performed for 13 PCGs and 2 rRNA genes. Based on the saturation plots (Figure S1), *atp8*, the third codon positions of all PCGs and the two rRNA genes were saturated and, thus, were excluded from the NT data set. The AA dataset contained amino acids translated from the 13 PCGs. The maximum likelihood (ML) and Bayesian inference (BI) trees generated from the NT dataset and AA dataset were mostly congruent, with subtle disparities in the support values of several branch nodes (Figure 8 and Figure 9).

The phylogenetic trees revealed the evolutionary relationships for the atyids within Caridea. At the species level, the three newly sequenced *N. denticulata* shrimps were closely grouped with *Neocaridina* species with strong nodal support (BP & BPP ≥ 95 & 0.95) (Figure 8 and Figure 9). Subsequently, the *Neocaridina* group was clustered with *Caridina longshan*. At the genus level, the monophyly of the genera *Atyopsis*, *Caridina*, *Neocaridina*, *Stygiocaris* and *Typhlatya* was not supported. According to the phylogenetic trees, the atyid shrimps in this study were divided into two highly supported major branches. *Atyopsis*, *Caridina* and *Neocaridina* formed one major branch, with *Neocaridina* appearing as a subgroup within the *Caridina* genus. *Halocaridina*, *Halocaridinides*, *Paratya*, *Stygiocaris*, *Typhlatya* and *Typhlopatsa* were also grouped together as the other major branch. Among these genera, *Stygiocaris* and *Typhlopatsa* formed a sister lineage and were clustered with all *Typhlatya* species, except for *Typhlatya galapagensis*. *Halocaridina rubra*, *Typhlatya galapagensis*, *Halocaridinides fowleri* and *Paratya australiensis* each formed their own group with well-supported values (BP & BPP ≥ 93 & 0.98), and the relationships were represented as … + *Halocaridina rubra* + *Typhlatya galapagensis* + *Halocaridinides fowleri* + *Paratya australiensis* (Figure 8 and Figure 9).

At the family level, species from most families formed independent clades, demonstrating strong monophyly of these families. Tree topologies indicated that there are four major clades. Clade I represents the family Pandalidae. Clade II contains Alpheidae, Glyphocrangonidae, Hippolytidae and Palaemonidae. Glyphocrangonidae and Hippolytidae, as well as Alpheidae and Palaemonidae, each formed a sister group and finally merged together. Clade III also comprises four families, with Acanthephyridae and Oplophoridae forming a sister group, then clustering with Alvinocarididae, and lastly with Nematocarcinidae. Clade IV only includes Atyidae species and is well supported (Figure 8 and Figure 9).

### 3.5. Mitochondrial Gene Order and Rearrangements

**Figure 10:**
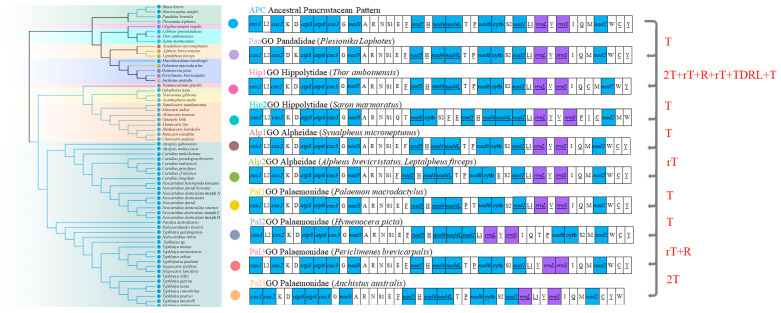
The ten mitochondrial gene order patterns of the studied Caridea species mapped onto the phylogenetic tree. Genes encoded by the light strand are underlined. The alternative rearrangement scenarios inferred from the putative ancestral pancrustacean pattern to the other nine distinct mitochondrial gene order patterns of the studied Caridea species through the CREx analysis.

A total of ten distinct patterns of mitochondrial gene order were identified in the fifty-nine available mitogenomes of caridean shrimps (Figure 10). Among them, the most widespread gene order pattern is the ancestral Pancrustacea pattern (ApcGO), which is shared by caridean shrimps belonging to nine families (Pandalidae, Glyphocrangonidae, Hippolytidae, Palaemonidae, Nematocarcinidae, Oplophoridae, Acanthephyridae, Alvinocarididae and Atyidae). In other words, the mitochondrial gene order in Atyidae is conserved. However, there are two or more mitochondrial gene order patterns within the same family, indicating that gene rearrangement in the mitogenomes of Caridea is prevalent. For instance, the species of Pandalidae and Alpheidae exhibit two gene orders (hereafter named ApcGO and PanGO; and Alp1GO and Alp2GO). Meanwhile, the species of Hippolytidae and Palaemonidae exhibit three and five gene order patterns (ApcGO, Hip1GO and Hip2GO; and ApcGO, Pal1GO, Pal2GO, Pal3GO and Pal4GO). Among these multiple arrangement patterns, a conserved gene block: *cox2*-*trnK*-*trnD*-*atp8*-*atp6*-*cox3*-*trnG*-*nad3*-*trnA*-*trnR*-*trnN*-*trnS^AGN^* was present across all mitogenomes of caridean shrimps. If only the 13 PCGs and two rRNA genes are considered, the gene order patterns of mitogenomes of caridean shrimps are almost identical, excluding the *Saron marmoratus* (Hip2GO) and *Hymenocera picta* (Pal2GO). Compared with the gene order of the ancestral Pancrustacea pattern, *S. marmoratus* has a translocation, for which the gene order is *nad5*-*nad4*-*nad4L*-*nad6*-*cytb* instead of *nad6*-*cytb*-*nad5*-*nad4*-*nad4L*, while the translocation of *nad1* was found in *H. picta*.

Based on the analysis of CREx, the rearrangement evolutionary pathways were inferred from the ancestral Pancrustacea pattern to the nine new gene order patterns included in this study, as shown in Figure 10. Most new gene order patterns (PanGO, Hip2GO, Alp1GO, Alp2GO, Pal1GO, Pal2GO, Pal3GO and Pal4GO) have been produced through transposition, reverse transposition and reversal events. Nevertheless, the Hip2GO is the most complicated one, resulting from successive events of transposition/reverse transposition, reversal, and TDRL (tandem duplication-random loss) (Figure 10).

## 4. Discussion

### 4.1. Morphological Differences

Kubo [52] conducted a detailed morphological comparison study of the atyid shrimps collected from Hiroshima (Japan), Shanghai (China) and Busan (Republic of Korea). Through statistical analysis, Kubo established a new genus, *Neocaridina*, based on the type species *Caridina denticulata* De Haan, 1849, which was separated from the genus *Caridina*. Compared with other species of atyid shrimps, *Neocaridina* exhibits stable and distinct morphological characteristics: the endopod of the first pair of pleopods in the male is notably widened, oval or subcircular, and the appendix masculina of the second pair of pleopods in the male is also widened and features relatively long setae.

Due to taxonomic difficulties, the genus *Neocaridina* has been constantly under revision and the validity of several species is currently questionable [8,9,11,14,15]. In Cai’s [8] revision of the genus *Neocaridina*, he described/redescribed eight subspecies of *N. denticulata*. However, most of these subspecies are not accepted today. To date, the two widely accepted subspecies of *N. denticulata* are *N. d. denticulata* and *N. d. sinensis*. *N. d. sinensis* was distinguished from *N. d. denticulata* by Kemp, 1918 mainly based on the rostral formula: 14-22/3-8 (vs. 10-15/2-5), and the anterior carpal margin of the first pereiopod exhibits a deep excavation, as opposed to slightly excavated. Nevertheless, in many cases, the rostral formula between the two subspecies is not significant, and the rostral length is more useful for distinguishing them, as proposed by Kubo [52]. Typically, the rostrum of *N. d. sinensis* does not extend beyond the end of the antennular peduncle, while it extends well beyond it in *N. d. denticulata*. Furthermore, sexual dimorphism of the last three pereiopods is present in *N. d. sinensis* but absent in *N. d. denticulata*. Moreover, Liang [13] included some of the *N. d. sinensis* described by Cai [8] in his new species *N. heteropoda*. Liang [3,13] also considered the distinguishing characters between *N. d. denticulata* and *N. d. sinensis* unreliable, and treated *N. d. sinensis* from China and Taiwan as a synonym of *N. d. denticulata*. Since neither Cai [8] nor Liang [3,13] had examined the type material of *N. d. sinensis* (which is probably lost), the definition of the above subspecies is rather uncertain for the time being.

Additionally, *N. davidi* Bouvier, 1904 has similar morphological features to *N. d. sinensis*, e.g., shorter rostrum which does not reach the end of the second segment of the antennular peduncle, deeply excavated carpus of first cheliped and significant sexual dimorphism observed in the third pair of pereiopods [8,10]. Consequently, Shih et al. [4,9] and Klotz et al. [10] suggested that *N. davidi* was synonymous with *N. d. sinensis*. Nevertheless, the validity of *N. davidi* remains questionable as it is uncertain whether Liang [3] examined the types of *N. davidi*. Moreover, he did not explain this synonymization in the text.

Across caridean shrimps, the rostrum shows large variations and is commonly used in the identification key of atyid shrimps. In previous taxonomic studies, the length of the rostrum was regarded as one of the morphological characteristics for the classification of the genus *Neocaridina*. *N. davidi* and *N. denticulata sinensis* have a shorter rostrum that does not reach the end of the third segment of the antennular peduncle [8,10], while *N. d. denticulata* has a long rostrum that exceeds the end of the antennular peduncle. In the present study, the rostrum of all specimens is nearly horizontal and showed slight differences in length, reaching or over-reaching the tip of the antennular peduncle. The slight differences in the length of the rostrum should be normal intraspecific variation. The confusion pertaining to the taxonomy of the genus *Neocaridina* has been, in part, due to natural variability in traditionally retained morphological characters that have been used for species identification. Furthermore, based on the research of cave shrimps of the genus *Troglocaris*, Jugovic et al. [53] also suggested that the rostral shape might not be a reliable taxonomic character in some generic and numerous specific diagnoses within Atyidae. One of the most representative instances is the intertidal marine shrimp, *Hippolyte sapphica*, which exhibits a unique and sharp rostral dimorphism: morphotype A featuring a well-developed dentate rostrum, while morphotype B with a short and juvenile-like toothless rostrum. Previous studies have demonstrated that both morphotypes belong to the same species and co-exist within the same habitat [54].

Except for the rostrum, *N. denticulata* shrimps of morph A, morph B and morph C exhibit continuous variation of the dactyli of the third pereiopod, and intraspecific variation exists in the length-width ratio of the endopod of the first pleopod and the length ratio of appendix masculine (Figure 2). *N. denticulata* demonstrates a preference for standing waters or slow water flow and usually avoids high current velocities, the submerged vegetation along the banks provides shelter for them [11,16,55]. This habitat preference emphasizes the wide range of habitats suitable for this generalist species. Furthermore, field and laboratory observations reveal that *N. denticulata* has a short reproductive cycle and is highly reproductive. In brief, these characteristics probably enable *N. denticulata* to have strong environmental tolerance, thus being able to establish populations at numerous sites and it has been reported as an introduced species in Hawaii, Germany, Poland and Hungary [10,12,56,57]. Correspondingly, the survival pressure and intraspecific competition are relatively low, resulting in diversified morphological characteristics.

Additionally, in this study, *N. denticulata* of morph C displays dimorphism in the third pereiopod dactyli, while morph A exhibits normal third pereiopod dactyli, and morph B exhibits an intermediate state between morph A and morph C (Figure 2). The sexual selection hypothesis suggests that if a certain feature of a species gives an advantage in competing with same-sex partners and is beneficial for improving mating success, this advantageous feature will be selected, leading to the development of sexual dimorphism. Sexual selection generally acts on males, making those with advantageous features more fit and, thus, strengthening these physical characteristics in constant same-sex competition [58,59]. The pereiopods of caridean shrimps play important roles in feeding, effectively defeating competitors for reproductive competition and enhancing mating success rates [60,61,62]. Compared with morph A and morph B, the third pereiopod dactyli of morph C is conspicuously broad, and several ventral marginal spines at the dactyli are thick, long and curved and slightly hook-shaped. Undoubtedly, larger and stronger pereiopods could help maintain its dominant position in the community.

### 4.2. Genetic Distances and Species Validity

According to the currently published mitogenomes for Atyidae, we calculated the genetic distances of 13 PCGs, *rrnS* and *rrnL* among different genera. The results indicate that the average genetic distance of those genes in the genus *Neocaridina* is significantly lower than that of the other four genera (*Neocaridina* 4.36% vs. *Atyopsis* 26%, *Caridina* 27.11%, *Stygiocaris* 20.01% and *Typhlatya* 28.59%) (Figure 6). And this fully demonstrates that there are many problems regarding the efficacy and diversity of species in the genus *Neocaridina*, and an urgent revision is necessary.

Shih and Cai [9] conducted an analysis of 16S rRNA and failed to distinguish *N. d. denticulata* (from Japan) and *N. d. sinensis* (from Taiwan and Hawaii) as they are identical or only 1 bp apart, and they might be the same species. More recently, the research of Shih et al. [4] indicated that *N. davidi* and *N. denticulata* are sister species with small interspecific distance. Furthermore, the genetic distance of 16S rRNA between the Israeli specimens of *N. denticulata* and those from Japan was analyzed. The results showed that, although there were morphological differences between them, the genetic distance between the groups did not exceed 1% [16]. Compared to other mitochondrial genes, 16S rRNA typically exhibits lower rates of substitution. In general, genetic divergence of no more than 1% in the 16S rRNA gene indicates conspecificity for decapod crustaceans [63,64]. That is to say, even if there are morphological differences, the Israeli specimens of *N. denticulata* are genetically related to Japanese populations and may have originated from the same source [16].

Moreover, it is notable that the phylogenetic relationships among several particular species of the genus *Neocaridina* remain ambiguous, and specimens that were identified as *N. d. denticulata, N. d. sinensis*, *N. heteropoda*, and *N. davidi* were also found to be in the same cluster [4,9,16]. Our phylogenetic analysis results agree with this (Figure 8 and Figure 9).

Additionally, another significant aspect is that as an ornamental shrimp, the *Neocaridina* shrimp is highly reproductive, environmentally tolerant and has become very popular in the aquarium industry as pet and aquatic plant cleaners. In order to achieve various colored specimens, there is constant crossbreeding of *Neocaridina* shrimp. This might lead to the variation in morphological characters for these shrimps, thereby further exacerbating the difficulties in taxonomy.

In summary, based on the analyses of the morphology and molecules conducted by numerous scholars [3,4,9,13,16] and combined with our research results, we contend that the *N. denticulata* complex has been overclassified. And there is a considerable likelihood of the occurrence of synonyms. *N. davidi*, *N. d. denticulata*, *N. d. sinensis* and *N. heteropoda* may belong to the same species. Moreover, *N. denticulata* was the first to be named and described, therefore we propose retaining the name *N. denticulata*.

The average genetic divergence of 16S rRNA and COI for the three newly determined *N. denticulate* mitogenomes (morph A, morph B and morph C) was 1.2 ± 0.2% and 3.1 ± 0.3%, respectively (Figure 7). For 16S rRNA, this value was nearly half of the average intrageneric genetic divergence (2.1 ± 0.3%) of *Neocaridina* and was significantly lower than the average intrageneric genetic divergences of other genera within the family of Atyidae (*Atyopsis* 20.9%, *Caridina* 22.80%, *Stygiocaris* 29.30% and *Typhlatya* 15.60%) (Figure 6). Research on the DNA barcoding of Crustacea has shown that the COI gene can effectively distinguish most closely related species and is frequently utilized as a standard barcoding gene in various crustacean taxa [65]. The best threshold for distinguishing intra- from interspecific divergence has initially been proposed to be about 3% in sequence divergence [66], but this value was subsequently modified approximately ten times, leading to changes in the mean intraspecific difference [67]. For Crustacea, the best threshold obtained by Lefébure et al. [65] for their broad crustacean corrected dataset was 16%. Moreover, a threshold of 0.18 substitutions per site would differentiate intraspecific variation within Palaemonidae [68]. Many studies have indicated that a species should fulfill two criteria: monophyly and distinctness [15,69]. In the present study, the average intrageneric divergence of COI in *Neocaridina* (4.7%, Figure 6) was only 1.5 times that of the intraspecific genetic distance (3.1%), and therefore, the *N. denticulata* shrimps (morph A, morph B and morph C) collected from the freshwater of Baiyangdian drainage area are recognized to belong to the same species, *N. denticulata*.

### 4.3. Mitogenomic Phylogeny

Atyids are the most species-rich freshwater shrimps. Owing to the fact that most authors who identified and described new species among the atyid shrimps have failed to investigate their relations with those already known, the family Atyidae contains a considerable number of ill-defined species and genera [70]. Hence, despite their widespread distribution and ecological importance, the phylogenetic relationships of atyid freshwater shrimps remain largely unresolved.

In previous studies, the phylogeny within the atyid shrimps was reconstructed based on morphological characters. Nevertheless, many of the taxonomic characters utilized can be highly variable, particularly when studying larger populations [53,71,72]. With the progress of sequencing technology, an increasing amount of molecular data is used for phylogenetic analysis, which has resulted in an unsatisfying state of atyid systematics, and the monophyly of several genera within the Atyidae has been challenged [17]. In this study, the atyid shrimps were divided into two highly supported major clades. *Atyopsis*, *Caridina* and *Neocaridina* formed one major clade, with *Neocaridina* emerging as a subgroup within the *Caridina* genus. *Halocaridina Halocaridinides*, *Paratya*, *Stygiocaris*, *Typhlatya* and *Typhlopatsa* were also grouped together as the other major clade.

The phylogenetic tree inferred from mitogenomes indicated that the species within the *Caridina* genus were not completely clustered together (Figure 8 and Figure 9). Instead, the *Neocaridina* group was clustered with *Caridina longshan*, inhabiting the streams in the karst caves of Hunan, China. Subsequently, they were clustered with other species of the genus *Caridina*. This indicates that the genus *Caridina* is paraphyletic, which is not a new result. Studies using molecular data by previous authors have shown that the genus *Caridina* is not monophyletic [73], with individual species groups being the sister group of the cavernicolous genera *Marosina*, *Pycnisia* [53], *Elephantis* [74] and *Neocaridina* (in this study). Therefore, considering the current taxonomic complexity of the genus *Caridina*, several *Caridina*-subclades still await their assignment to new genera.

Additionally, the genus *Typhlatya* does not seem to be monophyletic, as genera *Halocaridina* Holthuis, 1963, *Stygiocaris* Holmes, 1900 and *Typhlopatsa* Holthuis, 1956 were nested within the *Typhlatya* species (Figure 8 and Figure 9). Moreover, *Halocaridinides* and *Paratya* were subsequently clustered with the aforementioned “*Typhlatya* group”. This genetic split among different *Typhlatya* species was already visible in Zakšek et al. [75] and von Rintelen et al. [70].

In summary, although atyid freshwater shrimps are distributed worldwide and play a crucial role in the functioning of freshwater ecosystems, their phylogenetic relationships remain largely unresolved. In this study, we used mitogenomes to investigate the phylogenetic relationships of some genera within the family of Atyidae. The results are similar to those of previous studies [70,76,77], which all indicate that the established classification of Atyidae demands substantial taxonomic revision at all taxonomic levels. von Rintelen et al. [70] proposed a new suprageneric systematization of atyids, especially in the most speciose genus, *Caridina*. Based on the existing research findings, we believe that this proposal is feasible.

Based on the nucleotide sequences (NT dataset) and amino acid sequences (AA dataset) of mitochondrial protein-coding genes, a phylogenetic analysis of the Caridea was performed using ML and BI methods, respectively. The topology of these trees is mostly congruent, with only subtle disparities in the support values of several branch nodes (Figure 8 and Figure 9). The monophyly of the Infraorder Caridea has been confirmed by numerous morphological and molecular phylogenetic studies [17,19,20,22]. In this study, the involved families, for which more than one taxon was examined, formed monophyletic groups with robust support in both ML and BI analyses (Figure 8 and Figure 9), which is highly congruent with previous studies [21,78,79].

Pandalidae represents a diverse caridean group distributed and inhabiting both shallow and deep waters. Our phylogenetic reconstruction based on mitogenomes retrieved topologies that were consistent with recent phylogenomic studies in supporting the monophyly of Pandalidae [78,79]. Additionally, Pandalidae has the closest relationship with the group (Glyphocrangonidae + Hippolytidae) + (Alpheidae + Palaemonidae). This result was consistent with the previous finding revealed by five nuclear genes [20], suggesting that the families Palaemonidae, Lysmatidae, Pandalidae, Alpheidae and Hippolytidae were clustered in one clade. Nevertheless, there is controversy among the families of Alpheidae, Hippolytidae and Palaemonidae. Our analyses suggested that the families of Alpheidae and Palaemonidae formed a sister group, which was in accordance with those of previous results [19,22,78,79]. Whereas in Li et al.′s study [20], there was a closer affinity between Alpheidae and Hippolytidae, revealing discrepancies that might be attributed to the heterogeneity of the samples used.

The tree topologies indicated that Acanthephyridae and Oplophoridae were sister groups, clustering with Alvinocarididae, and subsequently with Nematocarcinidae. These families constituted the deep-sea and hydrothermal vent branches and eventually merged with the shallow-water and freshwater shrimp Atyidae to form a larger clade (Figure 8 and Figure 9). The findings are consistent with recent molecular analyses [78,79]. It is notable that there is still some controversy regarding the evolutionary position of Atyidae. Specifically, our results are consistent with the recent phylogenomic studies in supporting Atyidae at a deeper base in Caridea [19,79]. However, both Bracken et al. [17] and Li et al. [20] regarded Atyidae as basal lineages within Caridea. The inconsistent results might be attributed to the heterogeneity of data and variations in sample sizes.

In light of the aforementioned research findings, the phylogenetic topology of Caridea still presents questions. Undeniably, this is mainly due to the fact that the current taxon coverage of Caridea species data in GenBank is rather limited and uneven. Some families, such as Acanthephyridae, Glyphocrangonidae, Hippolytidae, Nematocarcinidae and Oplophoridae, have insufficient molecular data. Therefore, a more comprehensive representation of Caridea in future studies will be necessary to reconstruct the natural evolutionary history of the Caridean shrimps.

## 5. Conclusions

In the present study, we collected several *Neocaridina* shrimps from the Baiyangdian drainage area. Based on integrative taxonomy (morphological characteristics, genetic variation and phylogenetic analysis), we contend that *N. davidi*, *N. denticulata denticulata*, *N. d. sinensis* and *N. heteropoda* should belong to the same species, and we propose retaining the name *N. denticulata*. The reconstructed mitogenomic phylogeny indicates that the monophyly of several genera within Atyidae has been challenged, suggesting that the established classification of the Atyidae requires substantial taxonomic revision at all taxonomic levels. Furthermore, the tree topologies support Atyidae at a deeper base within Caridea. More comprehensive taxon sampling is still needed to resolve the explicit internal relationships among Caridea.

## Figures and Tables

**Table 1:** Organization of the mitogenomes of *Neocaridina denticulata* morph A, morph B and morph C.

Gene	Strand	Position		Size	Codon			Intergenic Nucleotides
		From	To		Start	Stop	Anticodon	
*cox1*	H	1/1/1	1536/1536/1536	1536/1536/1536	ATG/ATG/ATG	TAA/TAA/TAA		0/0/0
*trnL2*	H	1539/1539/1539	1602/1602/1602	64/64/64			TAA/TAA/TAA	2/2/2
*cox2*	H	1604/1604/1604	2291/2291/2291	688/688/688	ATG/ATG/ATG	T--/T--/T--		1/1/1
*trnK*	H	2292/2292/2292	2359/2359/2359	68/68/68			TTT/TTT/TTT	0/0/0
*trnD*	H	2365/2368/2368	2431/2435/2435	67/68/68			GTC/GTC/GTC	5/8/8
*atp8*	H	2432/2436/2436	2590/2594/2594	159/159/159	ATC/ATT/ATT	TAA/TAA/TAA		0/0/0
*atp6*	H	2584/2588/2588	3258/3262/3262	675/675/675	ATG/ATG/ATG	TAA/TAA/TAA		−7/−7/−7
*cox3*	H	3258/3262/3262	4043/4047/4047	786/786/786	ATG/ATG/ATG	TAG/TAG/TAG		−1/−1/−1
*trnG*	H	4047/4051/4051	4110/4115/4115	64/65/65			TCC/TCC/TCC	3/3/3
*nad3*	H	4111/4116/4116	4464/4469/4469	354/354/354	ATC/ATC/ATC	TAA/TAA/TAA		0/0/0
*trnA*	H	4463/4468/4468	4526/4531/4531	64/64/64			TGC/TGC/TGC	−2/−2/−2
*trnR*	H	4527/4532/4532	4589/4594/4594	63/63/63			TCG/TCG/TCG	0/0/0
*trnN*	H	4594/4599/4599	4660/4665/4665	67/67/67			GTT/GTT/GTT	4/4/4
*trnS1*	H	4661/4666/4666	4727/4732/4732	67/67/67			TCT/TCT/TCT	0/0/0
*trnE*	H	4728/4733/4733	4795/4800/4800	68/68/68			TTC/TTC/TTC	0/0/0
*trnF*	L	4794/4799/4799	4859/4864/4864	66/66/66			GAA/GAA/GAA	−2/−2/−2
*nad5*	L	4860/4865/4865	6587/6592/6592	1728/1728/1728	ATG/ATG/ATG	TAA/TAA/TAA		0/0/0
*trnH*	L	6588/6593/6593	6653/6658/6658	66/66/66			GTG/GTG/GTG	0/0/0
*nad4*	L	6654/6659/6659	7992/7997/7997	1339/1339/1339	ATG/ATG/ATG	T--/T--/T--		0/0/0
*nad4l*	L	7986/7991/7991	8288/8293/8293	303/303/303	ATG/ATG/ATG	TAA/TAA/TAA		−7/−7/−7
*trnT*	H	8291/8296/8296	8356/8361/8361	66/66/66			TGT/TGT/TGT	2/2/2
*trnP*	L	8357/8362/8362	8422/8427/8426	66/66/65			TGG/TGG/TGG	0/0/0
*nad6*	H	8425/8430/8429	8940/8945/8944	516/516/516	ATT/ATT/ATT	TAA/TAA/TAA		2/2/2
*cob*	H	8940/8945/8944	10076/10081/10080	1137/1137/1137	ATG/ATG/ATG	TAG/TAG/TAG		−1/−1/−1
*trnS2*	H	10075/10080/10079	10144/10149/10148	70/70/70			TGA/TGA/TGA	−2/−2/−2
*nad1*	L	10163/10168/10167	11104/11109/11108	942/942/942	ATT/ATT/ATT	TAA/TAA/TAA		18/18/18
*trnL1*	L	11129/11134/11133	11196/11200/11199	68/67/67			TAG/TAG/TAG	24/24/24
*rrnL*	L	11197/11201/11200	12530/12534/12530	1334/1334/1331				0/0/0
*trnV*	L	12531/12535/12531	12597/12601/12597	67/67/67			TAC/TAC/TAC	0/0/0
*rrnS*	L	12598/12602/12598	13458/13463/13459	861/862/862				0/0/0
*CR*	H	13459/13464/13460	14137/14142/14137	679/679/678				0/0/0
*trnI*	H	14138/14143/14138	14202/14207/14202	65/65/65			GAT/GAT/GAT	0/0/0
*trnQ*	L	14211/14216/14211	14278/14283/14278	68/68/68			TTG/TTG/TTG	8/8/8
*trnM*	H	14284/14289/14284	14349/14354/14349	66/66/66			CAT/CAT/CAT	5/5/5
*nad2*	H	14350/14355/14350	15354/15359/15354	1005/1005/1005	ATT/ATT/ATT	TAA/TAA/TAA		0/0/0
*trnW*	H	15353/15358/15353	15422/15427/15423	70/70/71			TCA/TCA/TCA	−2/−2/−2
*trnC*	L	15422/15427/15423	15486/15491/15487	65/65/65			GCA/GCA/GCA	−1/−1/−1
*trnY*	L	15487/15492/15488	15553/15558/15554	67/67/67			GTA/GTA/GTA	0/0/0

## Data Availability

The datasets generated for this study can be found in the GenBank (http://www.ncbi.nlm.nih.gov/genbank (accessed on 17 October 2024)).

## Supplementary Materials

The following supporting information can be downloaded at: https://www.mdpi.com/article/10.3390/cimb46110729/s1, Figure S1: Substitution saturation plots per codon position for 13 PCGs, 12S rRNA (*rrnS*) and 16S rRNA (*rrnL*); Table S1: The published mitogenomes information of Atyidae; Table S2: Amino acids usage of the *N. denticulata* (morph A, morph B and morph C); Table S3: The genetic divergence of 13PCGs, 12S rRNA (*rrnS*) and 16S rRNA (*rrnL*), respectively, within different genera of species in the Atyidae family; Table S4: The mitogenomes sequences used for phylogenetic reconstructions; Table S5: Best partitioning scheme and substitution models selected by PartitionFinder in this study.
