# Supplementary material for: Integrative Taxonomy Reveals New Insights into the Species Validity of the Neocaridina davidi-N. denticulata-N. heteropoda Complex and Mitogenomic Phylogeny of Caridean Shrimps
Source: Curr Issues Mol Biol. 2024 Oct 31;46(11):12279–98. doi: 10.3390/cimb46110729 (PMC11593339; doi:10.3390/cimb46110729)
Supplement: Supplementary file 1 [file cimb-46-00729-s001.zip › Table S4.pdf]

Table S4. The mitogenomes sequences used for phylogenetic reconstructions.

Order	Suborder	Infraorder	Superfamily	Family	Species	GenBank accession number	Sequence length (bp)
Decapoda	Pleocyemata	Caridea	Alpheoidea	Alpheidae	*Alpheus brevicristatus*	NC_079948	15,705
					Leptalpheus forceps	MN732884	15,463
					Synalpheus microneptunus	NC_047307	15,603
				Hippolytidae	*Lebbeus groenlandicus*	NC_045223	17,399
					Saron marmoratus	NC_050677	16,330
					Thor amboinensis	NC_051930	15,553
			Bresilioidea	Alvinocarididae	*Alvinocaris kexueae*	MH714459	15,864
					Chorocaris paulexa	MK309612	15,909
					Manuscaris liui	MH714461	15,903
					Mirocaris indica	NC_054368	15,922
					Nautilocaris saintlaurentae	KF226726	15,928
					Opaepele loihi	JQ035657	15,905
					Rimicaris variabilis	MN719306	15,909
					Shinkaicaris leurokolos	MF627741	15,903
			Atyoidea	Atyidae	*Atyopsis gabonensis*	OP650929	15,978
					Atyopsis moluccensis	OP618117	15,933
					Caridina gracilipes	NC_024751	15,550
					Caridina indistincta	NC_039593	15,461
					Caridina longshan	OP177695	16,853
					Caridina multidentata	NC_038067	15,825

Caridina cf. nilotica	NC_030219	15,479
Caridina pseudogracilirostris	NC_079936	15,451
Halocaridina rubra	NC_008413	16,065
Halocaridinides fowleri	NC_035412	15,977
Neocaridina davidi	MN418055	15,564
Neocaridina davidi koreana	MK907783	15,558
Neocaridina heteropoda koreana	NC_043865	15,558
Neocaridina denticulata	NC_023823	15,561
Neocaridina denticulata sinensis	MW238411	15,555
Neocaridina denticulate morph A	PQ246621	15,553
Neocaridina denticulate morph B	PQ246622	15,558
Neocaridina denticulate morph C	PQ246623	15,554
Paratya australiensis	NC_027603	15,990
Stygiocaris lancifera	NC_035404	15,787
Stygiocaris stylifera	NC_035411	15,812
Typhlatya arfeae	NC_035410	15,887
Typhlatya consobrina	NC_035407	15,785
Typhlatya dzilamensis	NC_035408	15,892
Typhlatya galapagensis	NC_035402	16,430
Typhlatya garciai	NC_035409	15,318
Typhlatya iliffei	NC_035401	15,926
Typhlatya miravetensis	NC_036335	15,865
Typhlatya mitchelli	NC_035403	15,814
Typhlatya monae	NC_035405	16,007
Typhlatya pearsei	KX844709	15,798

				Typhlatya sp. JR2016	KX844713	15,870
				Typhlatya taina	NC_035399	15,790
				Typhlopatsa pauliani	NC_035406	15,824
			Glyphocrangonidae	*Glyphocrangon regalis*	OP650930	15,918
		Nematocarcinoidea	Nematocarcinidae	*Nematocarcinus gracilis*	MH714456	15,919
		Oplophoroidea	Oplophoridae	*Oplophorus typus*	MK330597	16,880
			Acanthephyridae	*Notostomus gibbosus*	NC_059935	17,590
				Acanthephyra smithi	MH714455	17,165
		Palaemonoidea	Palaemonidae	*Anchistus australis*	NC_046034	15,396
				Hymenocera picta	NC_039631	15,786
				Macrobrachium rosenbergii	OM289965	15,766
				Palaemon macrodactylus	OQ512152	15,777
				Periclimenes brevicarpalis	OL752710	16,673
		Pandaloidea	Pandalidae	*Bitias brevis*	MG674229	15,891
				Heterocarpus ensifer	MG674228	15,939
				Pandalus borealis	LC341266	15,956
				Plesionika lophotes	NC_072243	15,933
	Dendrobranchiata	Penaeoidea	Aristeidae	*Aristeus virilis*	MG582605	15,936
			Penaeidae	*Penaeus chinensis*	DQ656600	16,009
Stomatopoda	Unipeltata	Squilloidea	Squillidae	*Oratosquilla oratoria*	GQ292769	15,783
		Lysiosquilloidea	Lysiosquillidae	*Lysiosquillina maculata*	NC_007443	16,325
