# Supplementary figures and images for: Integrative Taxonomy Reveals New Insights into the Species Validity of the Neocaridina davidi-N. denticulata-N. heteropoda Complex and Mitogenomic Phylogeny of Caridean Shrimps
Source: Curr Issues Mol Biol. 2024 Oct 31;46(11):12279–98. doi: 10.3390/cimb46110729 (PMC11593339; doi:10.3390/cimb46110729)

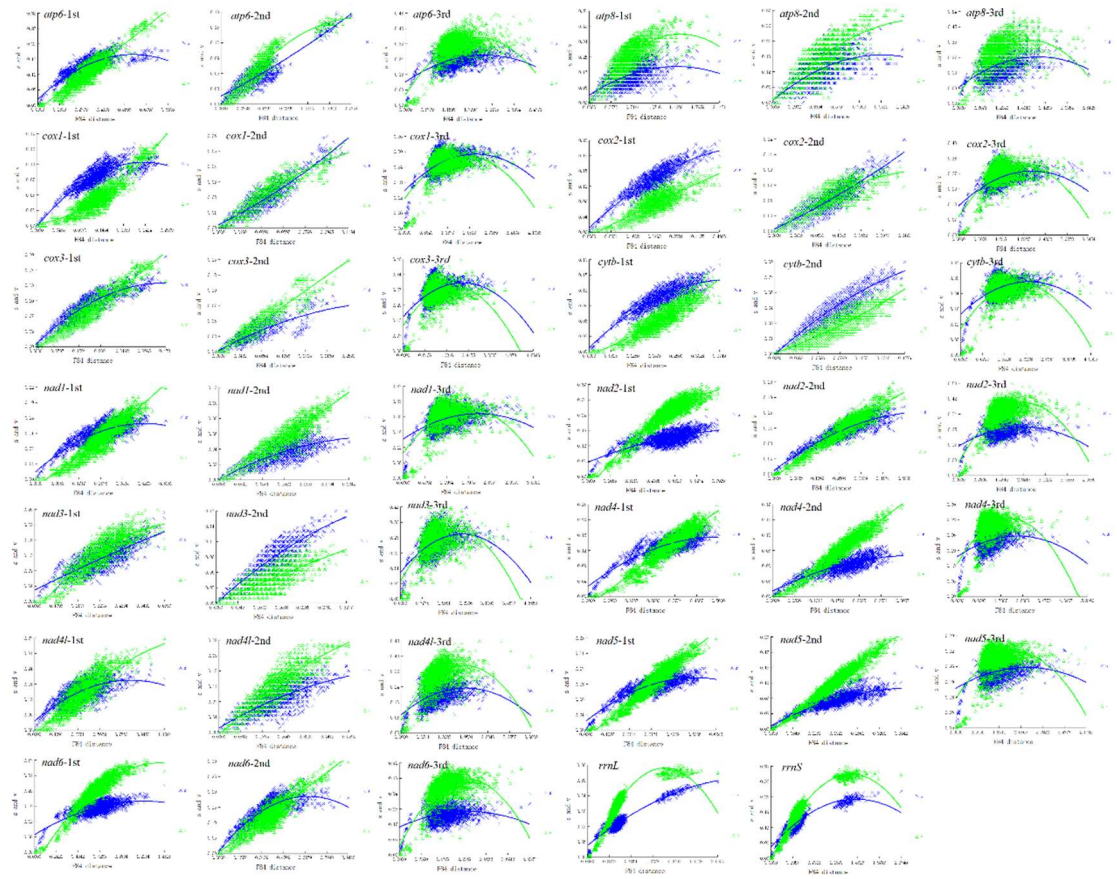

**Figure S1.** Substitution saturation plots per codon position for 13 PCGs, *rrnL* and *rrnS*.

Supplement: Supplementary file 1 [file cimb-46-00729-s001.zip › Figure S1.pdf]
